# Book Reviews

**Published:** 1870-11

**Authors:** 


### BIBLIOGRAPHICAL NOTICES.

---

*Deformities of the Mouth, Congenital and Acquired, with their Mechanical Treatment.* By James Oakley Coles, Honorary Dentist to Golden Square Hospital for Diseases of the Throat; Member of Odontological Society of Great Britain, etc. Second edition. Revised and enlarged. Publishers, Lindsay & Blackiston, Philadelphia.

This valuable contribution to dental literature, on the treatment of congenital and acquired defects of the palatal, is embellished with no less than twenty finely executed colored plates, and fifty-one wood cuts, and presents this subject in a comprehensive and impartial manner, credit being given throughout the work to all who have suggested improvements in methods of treatment. The author in describing his principles of treatment, remarks that it is based on the inventions of Dr. Kingsley, but considerably modified, and that much as he believes in the soundness of that system of curative dentistry, introduced by Dr. Kingsley, and followed out since by so many others besides himself, he is by no means wedded to his belief to such an extent as to undervalue the merits of surgical operation for congenital cleft palate. The work is dedicated to Sir William Fergusson, the eminent English surgeon, and will prove a valuable aid to all who are interested in the treatment of defects of the palate.

---

*Descriptive Catalogue of the New Sydenham Society's Atlas of Portraits of Diseases of the Skin.* By Jonathan Hutchinson, F. R. C. S. Part I. Lindsay & Blackiston, Philadelphia.

---

*The Physician's Visiting List for* 1871. Lindsay & Blackiston, publishers, Philadelphia. For several years past we have been using this work as a dental appointment book, and find that it answers the purpose admirably.

---

